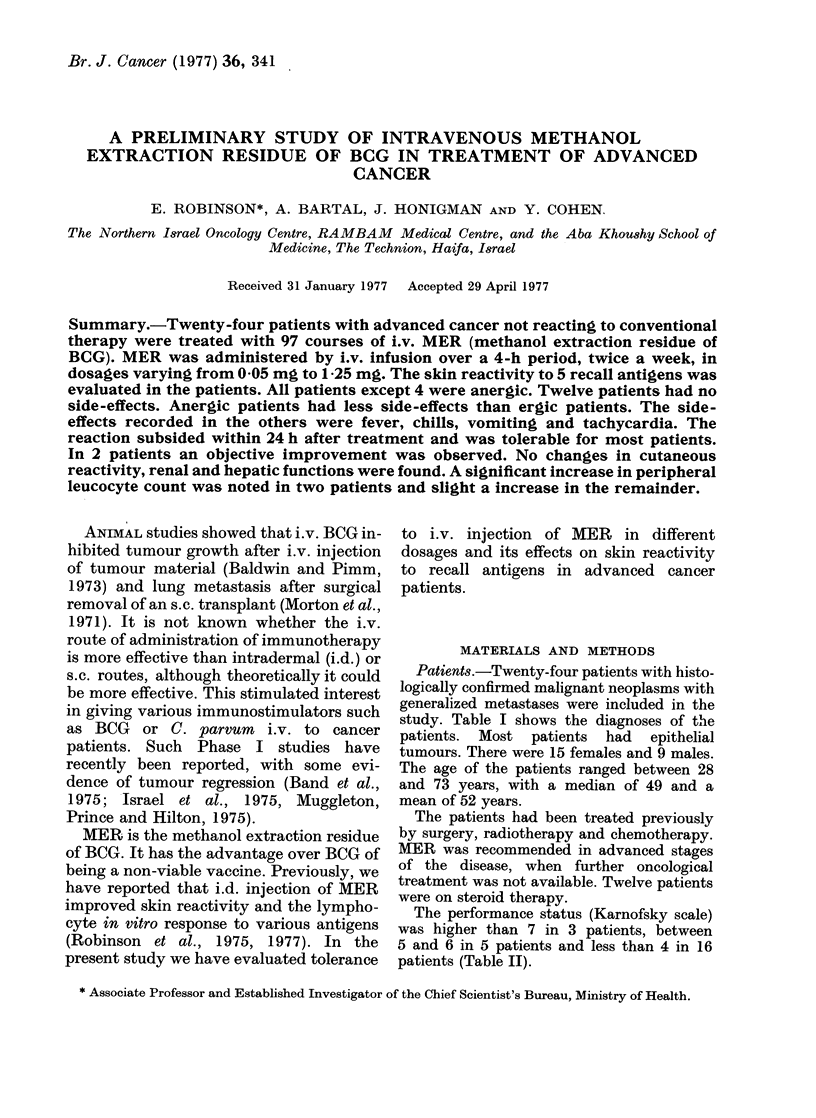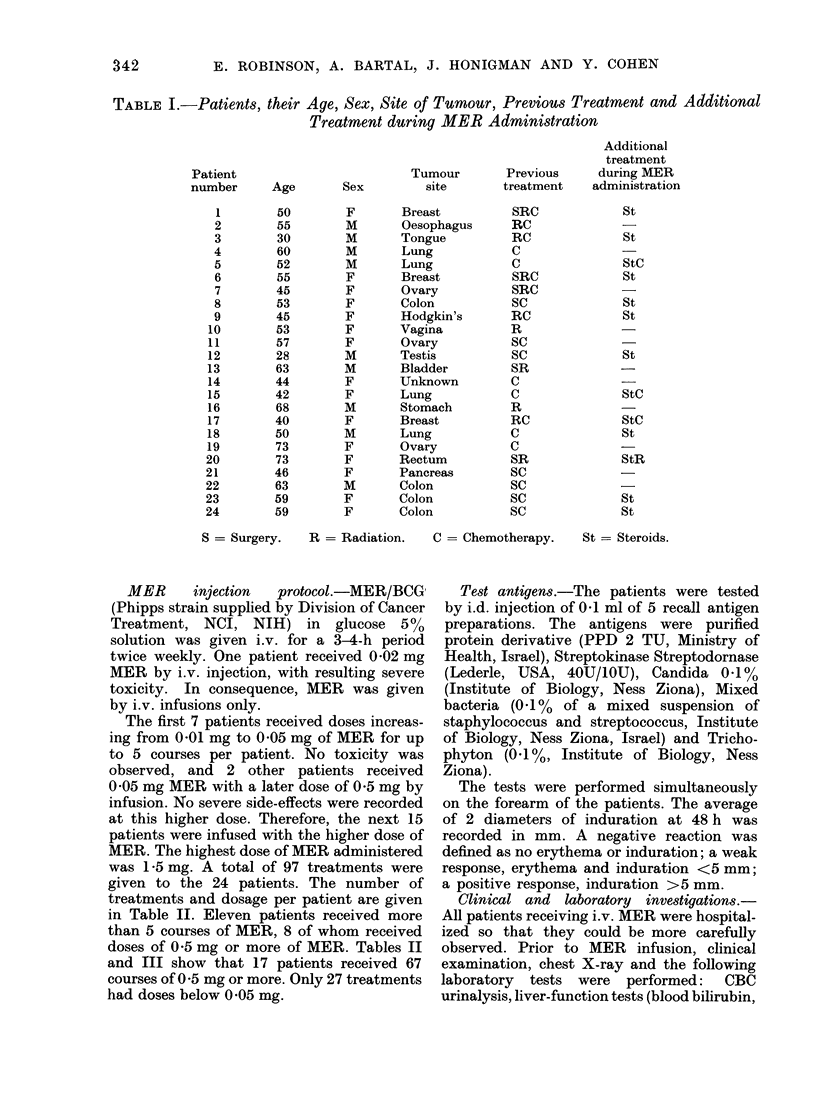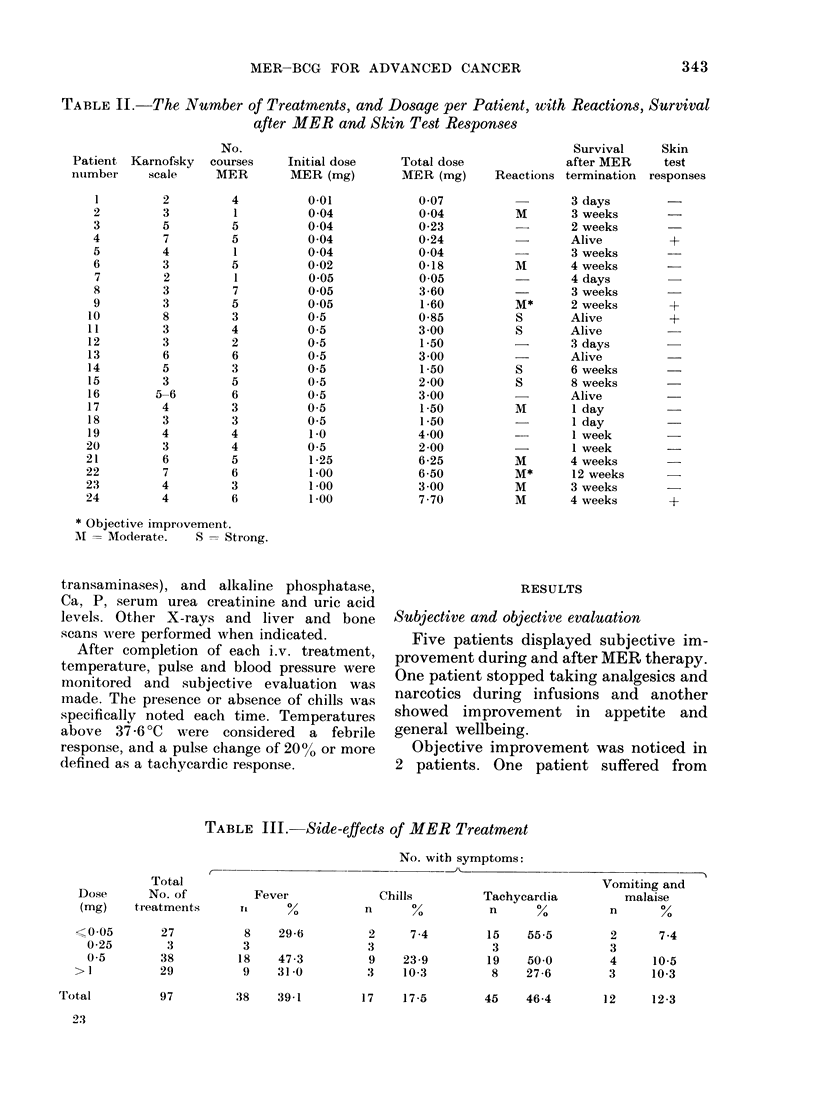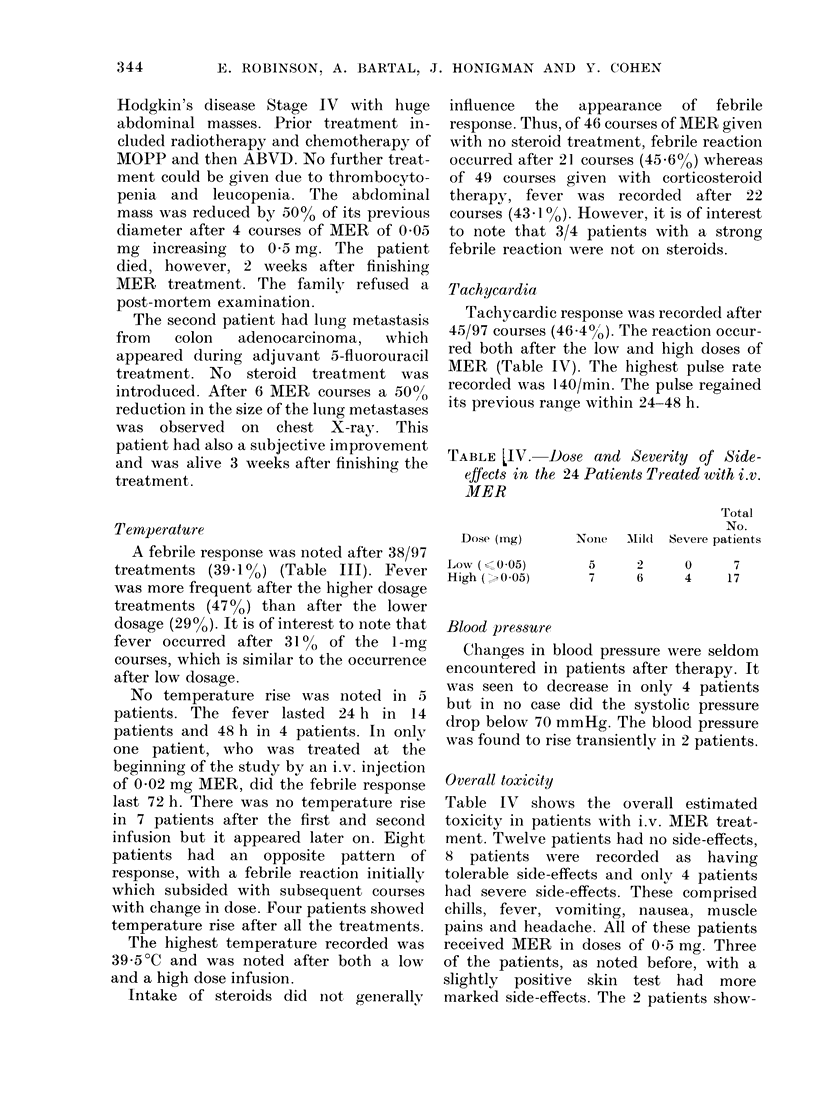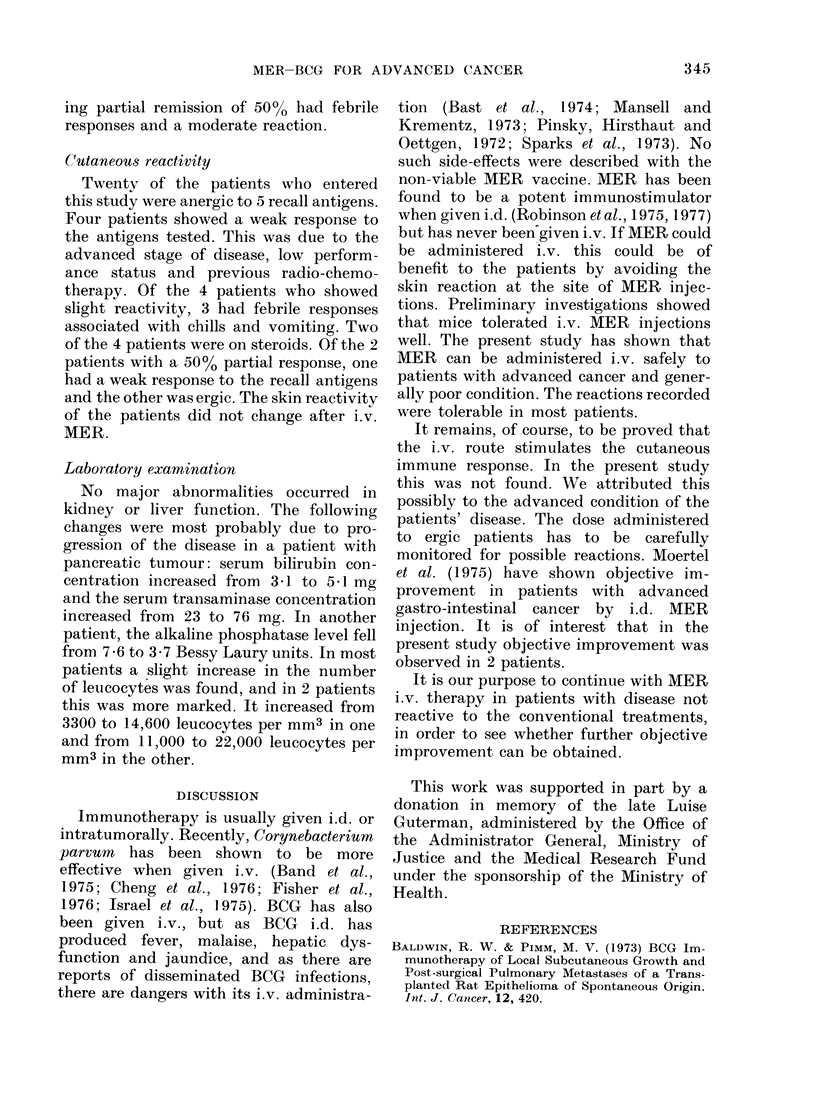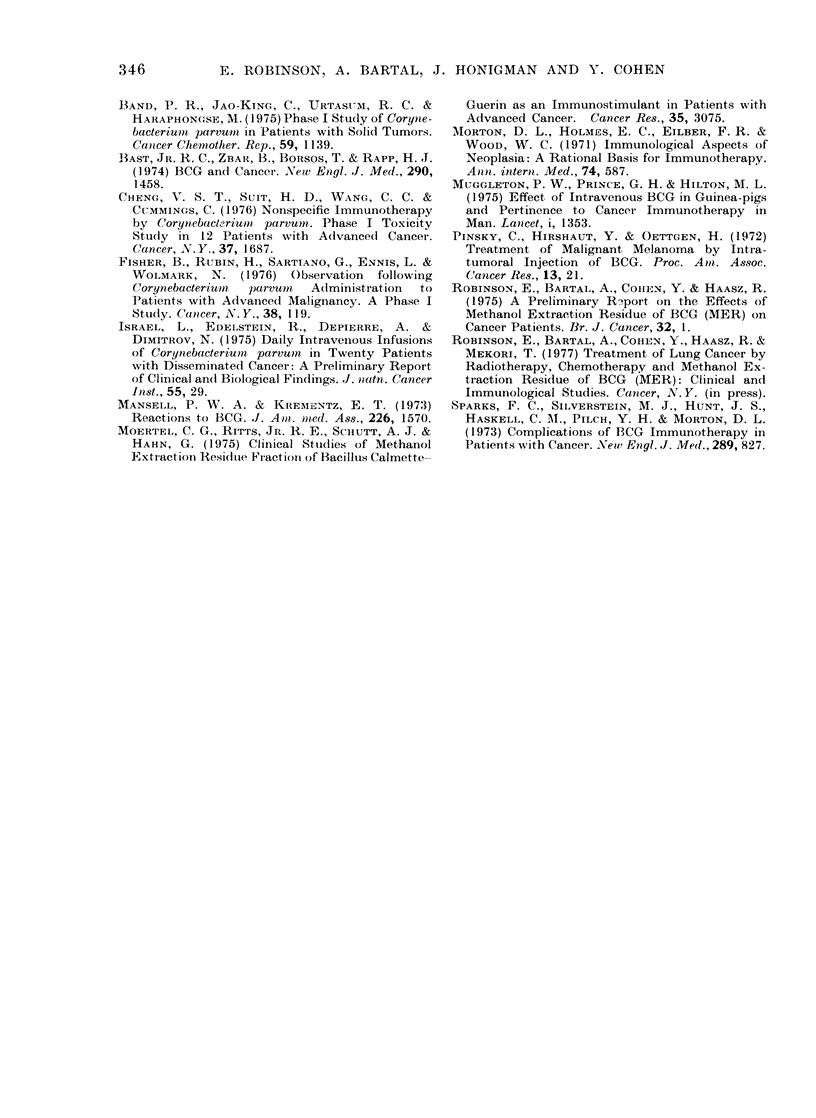# A preliminary study of intravenous methanol extraction residue of BCG in treatment of advanced cancer.

**DOI:** 10.1038/bjc.1977.198

**Published:** 1977-09

**Authors:** E. Robinson, A. Bartal, J. Honigman, Y. Cohen

## Abstract

Twenty-four patients with advanced cancer not reacting to conventional therapy were treated with 97 courses of i.v. MER (methanol extraction residue of BCG). MER was administered by i.v. infusion over a 4-h period, twice a week, in dosages varying from 0.05 mg to 1.25 mg. The skin reactivity to 5 recall antigens was evaluated in the patients. All patients except 4 were anergic. Twelve patients had no side-effects. Anergic patients had less side-effects than ergic patients. The side-effects recorded in the others were fever, chills, vomiting and tachycardia. The reaction subsided within 24 h after treatment and was tolerable for most patients. In 2 patients an objective improvement was observed. No changes in cutaneous reactivity, renal and hepatic functions were found. A significant increase in peripheral leucocyte count was noted in two patients and slight a increase in the remainder.


*Br. J. Cancer* (1977) **36**, 341

### A PRELIMINARY STUDY OF INTRAVENOUS METHANOL EXTRACTION RESIDUE OF BCG IN TREATMENT OF ADVANCED CANCER

E. ROBINSON*, A. BARTAL, J. HONIGMAN AND Y. COHEN

*The Northern Israel Oncology Centre, RAMBAM Medical Centre, and the Aba Khoushy School of Medicine, The Technion, Haifa, Israel*

Received 31 January 1977 Accepted 29 April 1977

**Summary.—Twenty-four patients with advanced cancer not reacting to conventional therapy were treated with 97 courses of i.v. MER (methanol extraction residue of BCG). MER was administered by i.v. infusion over a 4-h period, twice a week, in dosages varying from 0·05 mg to 1·25 mg. The skin reactivity to 5 recall antigens was evaluated in the patients. All patients except 4 were anergic. Twelve patients had no side-effects. Anergic patients had less side-effects than ergic patients. The side-effects recorded in the others were fever, chills, vomiting and tachycardia. The reaction subsided within 24 h after treatment and was tolerable for most patients. In 2 patients an objective improvement was observed. No changes in cutaneous reactivity, renal and hepatic functions were found. A significant increase in peripheral leucocyte count was noted in two patients and slight a increase in the remainder.**

ANIMAL studies showed that i.v. BCG inhibited tumour growth after i.v. injection of tumour material (Baldwin and Pimm, 1973) and lung metastasis after surgical removal of an s.c. transplant (Morton *et al.*, 1971). It is not known whether the i.v. route of administration of immunotherapy is more effective than intradermal (i.d.) or s.c. routes, although theoretically it could be more effective. This stimulated interest in giving various immunostimulators such as BCG or *C. parvum* i.v. to cancer patients. Such Phase I studies have recently been reported, with some evidence of tumour regression (Band *et al.*, 1975; Israel *et al.*, 1975, Muggleton, Prince and Hilton, 1975).

MER is the methanol extraction residue of BCG. It has the advantage over BCG of being a non-viable vaccine. Previously, we have reported that i.d. injection of MER improved skin reactivity and the lymphocyte *in vitro* response to various antigens (Robinson *et al.*, 1975, 1977). In the present study we have evaluated tolerance to i.v. injection of MER in different dosages and its effects on skin reactivity to recall antigens in advanced cancer patients.

### MATERIALS AND METHODS

*Patients*.—Twenty-four patients with histologically confirmed malignant neoplasms with generalized metastases were included in the study. Table I shows the diagnoses of the patients. Most patients had epithelial tumours. There were 15 females and 9 males. The age of the patients ranged between 28 and 73 years, with a median of 49 and a mean of 52 years.

The patients had been treated previously by surgery, radiotherapy and chemotherapy. MER was recommended in advanced stages of the disease, when further oncological treatment was not available. Twelve patients were on steroid therapy.

The performance status (Karnofsky scale) was higher than 7 in 3 patients, between 5 and 6 in 5 patients and less than 4 in 16 patients (Table II).

* Associate Professor and Established Investigator of the Chief Scientist's Bureau, Ministry of Health.

TABLE I.—*Patients, their Age, Sex, Site of Tumour, Previous Treatment and Additional Treatment during MER Administration*

| Patient number | Age | Sex | Tumour site | Previous treatment | Additional treatment during MER administration |
|---|---|---|---|---|---|
| 1 | 50 | F | Breast | SRC | St |
| 2 | 55 | M | Oesophagus | RC | — |
| 3 | 30 | M | Tongue | RC | St |
| 4 | 60 | M | Lung | C | — |
| 5 | 52 | M | Lung | C | StC |
| 6 | 55 | F | Breast | SRC | St |
| 7 | 45 | F | Ovary | SRC | — |
| 8 | 53 | F | Colon | SC | St |
| 9 | 45 | F | Hodgkin's | RC | St |
| 10 | 53 | F | Vagina | R | — |
| 11 | 57 | F | Ovary | SC | — |
| 12 | 28 | M | Testis | SC | St |
| 13 | 63 | M | Bladder | SR | — |
| 14 | 44 | F | Unknown | C | — |
| 15 | 42 | F | Lung | C | StC |
| 16 | 68 | M | Stomach | R | — |
| 17 | 40 | F | Breast | RC | StC |
| 18 | 50 | M | Lung | C | St |
| 19 | 73 | F | Ovary | C | — |
| 20 | 73 | F | Rectum | SR | StR |
| 21 | 46 | F | Pancreas | SC | — |
| 22 | 63 | M | Colon | SC | — |
| 23 | 59 | F | Colon | SC | St |
| 24 | 59 | F | Colon | SC | St |

S = Surgery. R = Radiation. C = Chemotherapy. St = Steroids.

*MER injection protocol.*—MER/BCG (Phipps strain supplied by Division of Cancer Treatment, NCI, NIH) in glucose 5% solution was given i.v. for a 3–4-h period twice weekly. One patient received 0·02 mg MER by i.v. injection, with resulting severe toxicity. In consequence, MER was given by i.v. infusions only.

The first 7 patients received doses increasing from 0·01 mg to 0·05 mg of MER for up to 5 courses per patient. No toxicity was observed, and 2 other patients received 0·05 mg MER with a later dose of 0·5 mg by infusion. No severe side-effects were recorded at this higher dose. Therefore, the next 15 patients were infused with the higher dose of MER. The highest dose of MER administered was 1·5 mg. A total of 97 treatments were given to the 24 patients. The number of treatments and dosage per patient are given in Table II. Eleven patients received more than 5 courses of MER, 8 of whom received doses of 0·5 mg or more of MER. Tables II and III show that 17 patients received 67 courses of 0·5 mg or more. Only 27 treatments had doses below 0·05 mg.

*Test antigens.*—The patients were tested by i.d. injection of 0·1 ml of 5 recall antigen preparations. The antigens were purified protein derivative (PPD 2 TU, Ministry of Health, Israel), Streptokinase Streptodornase (Lederle, USA, 40U/10U), Candida 0·1% (Institute of Biology, Ness Ziona), Mixed bacteria (0·1% of a mixed suspension of staphylococcus and streptococcus, Institute of Biology, Ness Ziona, Israel) and Trichophyton (0·1%, Institute of Biology, Ness Ziona).

The tests were performed simultaneously on the forearm of the patients. The average of 2 diameters of induration at 48 h was recorded in mm. A negative reaction was defined as no erythema or induration; a weak response, erythema and induration $<5$ mm; a positive response, induration $>5$ mm.

*Clinical and laboratory investigations.*—All patients receiving i.v. MER were hospitalized so that they could be more carefully observed. Prior to MER infusion, clinical examination, chest X-ray and the following laboratory tests were performed: CBC urinalysis, liver-function tests (blood bilirubin,

TABLE II.—*The Number of Treatments, and Dosage per Patient, with Reactions, Survival after MER and Skin Test Responses*

| Patient number | Karnofsky scale | No. courses MER | Initial dose MER (mg) | Total dose MER (mg) | Reactions | Survival after MER termination | Skin test responses |
|---|---|---|---|---|---|---|---|
| 1 | 2 | 4 | 0·01 | 0·07 | — | 3 days | — |
| 2 | 3 | 1 | 0·04 | 0·04 | M | 3 weeks | — |
| 3 | 5 | 5 | 0·04 | 0·23 | — | 2 weeks | — |
| 4 | 7 | 5 | 0·04 | 0·24 | — | Alive | + |
| 5 | 4 | 1 | 0·04 | 0·04 | — | 3 weeks | — |
| 6 | 3 | 5 | 0·02 | 0·18 | M | 4 weeks | — |
| 7 | 2 | 1 | 0·05 | 0·05 | — | 4 days | — |
| 8 | 3 | 7 | 0·05 | 3·60 | — | 3 weeks | — |
| 9 | 3 | 5 | 0·05 | 1·60 | M* | 2 weeks | + |
| 10 | 8 | 3 | 0·5 | 0·85 | S | Alive | + |
| 11 | 3 | 4 | 0·5 | 3·00 | S | Alive | — |
| 12 | 3 | 2 | 0·5 | 1·50 | — | 3 days | — |
| 13 | 6 | 6 | 0·5 | 3·00 | — | Alive | — |
| 14 | 5 | 3 | 0·5 | 1·50 | S | 6 weeks | — |
| 15 | 3 | 5 | 0·5 | 2·00 | S | 8 weeks | — |
| 16 | 5–6 | 6 | 0·5 | 3·00 | — | Alive | — |
| 17 | 4 | 3 | 0·5 | 1·50 | M | 1 day | — |
| 18 | 3 | 3 | 0·5 | 1·50 | — | 1 day | — |
| 19 | 4 | 4 | 1·0 | 4·00 | — | 1 week | — |
| 20 | 3 | 4 | 0·5 | 2·00 | — | 1 week | — |
| 21 | 6 | 5 | 1·25 | 6·25 | M | 4 weeks | — |
| 22 | 7 | 6 | 1·00 | 6·50 | M* | 12 weeks | — |
| 23 | 4 | 3 | 1·00 | 3·00 | M | 3 weeks | — |
| 24 | 4 | 6 | 1·00 | 7·70 | M | 4 weeks | + |

* Objective improvement.

M = Moderate. S = Strong.

transaminases), and alkaline phosphatase, Ca, P, serum urea creatinine and uric acid levels. Other X-rays and liver and bone scans were performed when indicated.

After completion of each i.v. treatment, temperature, pulse and blood pressure were monitored and subjective evaluation was made. The presence or absence of chills was specifically noted each time. Temperatures above 37·6 °C were considered a febrile response, and a pulse change of 20% or more defined as a tachycardic response.

### RESULTS

### *Subjective and objective evaluation*

Five patients displayed subjective improvement during and after MER therapy. One patient stopped taking analgesics and narcotics during infusions and another showed improvement in appetite and general wellbeing.

Objective improvement was noticed in 2 patients. One patient suffered from

TABLE III.—*Side-effects of MER Treatment*

| Dose (mg) | Total No. of treatments | No. with symptoms: Fever n | % | Chills n | % | Tachycardia n | % | Vomiting and malaise n | % |
|---|---|---|---|---|---|---|---|---|---|
| ≤0·05 | 27 | 8 | 29·6 | 2 | 7·4 | 15 | 55·5 | 2 | 7·4 |
| 0·25 | 3 | 3 | | 3 | | 3 | | 3 | |
| 0·5 | 38 | 18 | 47·3 | 9 | 23·9 | 19 | 50·0 | 4 | 10·5 |
| >1 | 29 | 9 | 31·0 | 3 | 10·3 | 8 | 27·6 | 3 | 10·3 |
| Total | 97 | 38 | 39·1 | 17 | 17·5 | 45 | 46·4 | 12 | 12·3 |

Hodgkin's disease Stage IV with huge abdominal masses. Prior treatment included radiotherapy and chemotherapy of MOPP and then ABVD. No further treatment could be given due to thrombocytopenia and leucopenia. The abdominal mass was reduced by 50% of its previous diameter after 4 courses of MER of 0·05 mg increasing to 0·5 mg. The patient died, however, 2 weeks after finishing MER treatment. The family refused a post-mortem examination.

The second patient had lung metastasis from colon adenocarcinoma, which appeared during adjuvant 5-fluorouracil treatment. No steroid treatment was introduced. After 6 MER courses a 50% reduction in the size of the lung metastases was observed on chest X-ray. This patient had also a subjective improvement and was alive 3 weeks after finishing the treatment.

*Temperature*

A febrile response was noted after 38/97 treatments (39·1%) (Table III). Fever was more frequent after the higher dosage treatments (47%) than after the lower dosage (29%). It is of interest to note that fever occurred after 31% of the 1-mg courses, which is similar to the occurrence after low dosage.

No temperature rise was noted in 5 patients. The fever lasted 24 h in 14 patients and 48 h in 4 patients. In only one patient, who was treated at the beginning of the study by an i.v. injection of 0·02 mg MER, did the febrile response last 72 h. There was no temperature rise in 7 patients after the first and second infusion but it appeared later on. Eight patients had an opposite pattern of response, with a febrile reaction initially which subsided with subsequent courses with change in dose. Four patients showed temperature rise after all the treatments.

The highest temperature recorded was 39·5 °C and was noted after both a low and a high dose infusion.

Intake of steroids did not generally influence the appearance of febrile response. Thus, of 46 courses of MER given with no steroid treatment, febrile reaction occurred after 21 courses (45·6%) whereas of 49 courses given with corticosteroid therapy, fever was recorded after 22 courses (43·1%). However, it is of interest to note that 3/4 patients with a strong febrile reaction were not on steroids.

*Tachycardia*

Tachycardic response was recorded after 45/97 courses (46·4%). The reaction occurred both after the low and high doses of MER (Table IV). The highest pulse rate recorded was 140/min. The pulse regained its previous range within 24–48 h.

TABLE IV.—*Dose and Severity of Side-effects in the 24 Patients Treated with i.v. MER*

| Dose (mg) | None | Mild | Severe | Total No. patients |
|---|---|---|---|---|
| Low ($\leqslant$0·05) | 5 | 2 | 0 | 7 |
| High ($\geqslant$0·05) | 7 | 6 | 4 | 17 |

*Blood pressure*

Changes in blood pressure were seldom encountered in patients after therapy. It was seen to decrease in only 4 patients but in no case did the systolic pressure drop below 70 mmHg. The blood pressure was found to rise transiently in 2 patients.

*Overall toxicity*

Table IV shows the overall estimated toxicity in patients with i.v. MER treatment. Twelve patients had no side-effects, 8 patients were recorded as having tolerable side-effects and only 4 patients had severe side-effects. These comprised chills, fever, vomiting, nausea, muscle pains and headache. All of these patients received MER in doses of 0·5 mg. Three of the patients, as noted before, with a slightly positive skin test had more marked side-effects. The 2 patients showing partial remission of 50% had febrile responses and a moderate reaction.

*Cutaneous reactivity*

Twenty of the patients who entered this study were anergic to 5 recall antigens. Four patients showed a weak response to the antigens tested. This was due to the advanced stage of disease, low performance status and previous radio-chemotherapy. Of the 4 patients who showed slight reactivity, 3 had febrile responses associated with chills and vomiting. Two of the 4 patients were on steroids. Of the 2 patients with a 50% partial response, one had a weak response to the recall antigens and the other was ergic. The skin reactivity of the patients did not change after i.v. MER.

*Laboratory examination*

No major abnormalities occurred in kidney or liver function. The following changes were most probably due to progression of the disease in a patient with pancreatic tumour: serum bilirubin concentration increased from 3·1 to 5·1 mg and the serum transaminase concentration increased from 23 to 76 mg. In another patient, the alkaline phosphatase level fell from 7·6 to 3·7 Bessy Laury units. In most patients a slight increase in the number of leucocytes was found, and in 2 patients this was more marked. It increased from 3300 to 14,600 leucocytes per $mm^3$ in one and from 11,000 to 22,000 leucocytes per $mm^3$ in the other.

### DISCUSSION

Immunotherapy is usually given i.d. or intratumorally. Recently, *Corynebacterium parvum* has been shown to be more effective when given i.v. (Band *et al.*, 1975; Cheng *et al.*, 1976; Fisher *et al.*, 1976; Israel *et al.*, 1975). BCG has also been given i.v., but as BCG i.d. has produced fever, malaise, hepatic dysfunction and jaundice, and as there are reports of disseminated BCG infections, there are dangers with its i.v. administration (Bast *et al.*, 1974; Mansell and Krementz, 1973; Pinsky, Hirsthaut and Oettgen, 1972; Sparks *et al.*, 1973). No such side-effects were described with the non-viable MER vaccine. MER has been found to be a potent immunostimulator when given i.d. (Robinson *et al.*, 1975, 1977) but has never been given i.v. If MER could be administered i.v. this could be of benefit to the patients by avoiding the skin reaction at the site of MER injections. Preliminary investigations showed that mice tolerated i.v. MER injections well. The present study has shown that MER can be administered i.v. safely to patients with advanced cancer and generally poor condition. The reactions recorded were tolerable in most patients.

It remains, of course, to be proved that the i.v. route stimulates the cutaneous immune response. In the present study this was not found. We attributed this possibly to the advanced condition of the patients' disease. The dose administered to ergic patients has to be carefully monitored for possible reactions. Moertel *et al.* (1975) have shown objective improvement in patients with advanced gastro-intestinal cancer by i.d. MER injection. It is of interest that in the present study objective improvement was observed in 2 patients.

It is our purpose to continue with MER i.v. therapy in patients with disease not reactive to the conventional treatments, in order to see whether further objective improvement can be obtained.

This work was supported in part by a donation in memory of the late Luise Guterman, administered by the Office of the Administrator General, Ministry of Justice and the Medical Research Fund under the sponsorship of the Ministry of Health.

### REFERENCES

Baldwin, R. W. & Pimm, M. V. (1973) BCG Immunotherapy of Local Subcutaneous Growth and Post-surgical Pulmonary Metastases of a Transplanted Rat Epithelioma of Spontaneous Origin. *Int. J. Cancer*, **12**, 420.

BAND, P. R., JAO-KING, C., URTASUM, R. C. & HARAPHONGSE, M. (1975) Phase I Study of *Corynebacterium parvum* in Patients with Solid Tumors. *Cancer Chemother. Rep.*, **59**, 1139.

BAST, JR. R. C., ZBAR, B., BORSOS, T. & RAPP, H. J. (1974) BCG and Cancer. *New Engl. J. Med.*, **290**, 1458.

CHENG, V. S. T., SUIT, H. D., WANG, C. C. & CUMMINGS, C. (1976) Nonspecific Immunotherapy by *Corynebacterium parvum*. Phase I Toxicity Study in 12 Patients with Advanced Cancer. *Cancer, N.Y.*, **37**, 1687.

FISHER, B., RUBIN, H., SARTIANO, G., ENNIS, L. & WOLMARK, N. (1976) Observation following *Corynebacterium parvum* Administration to Patients with Advanced Malignancy. A Phase I Study. *Cancer, N.Y.*, **38**, 119.

ISRAEL, L., EDELSTEIN, R., DEPIERRE, A. & DIMITROV, N. (1975) Daily Intravenous Infusions of *Corynebacterium parvum* in Twenty Patients with Disseminated Cancer: A Preliminary Report of Clinical and Biological Findings. *J. natn. Cancer Inst.*, **55**, 29.

MANSELL, P. W. A. & KREMENTZ, E. T. (1973) Reactions to BCG. *J. Am. med. Ass.*, **226**, 1570.

MOERTEL, C. G., RITTS, JR. R. E., SCHUTT, A. J. & HAHN, G. (1975) Clinical Studies of Methanol Extraction Residue Fraction of Bacillus Calmette–Guerin as an Immunostimulant in Patients with Advanced Cancer. *Cancer Res.*, **35**, 3075.

MORTON, D. L., HOLMES, E. C., EILBER, F. R. & WOOD, W. C. (1971) Immunological Aspects of Neoplasia: A Rational Basis for Immunotherapy. *Ann. intern. Med.*, **74**, 587.

MUGGLETON, P. W., PRINCE, G. H. & HILTON, M. L. (1975) Effect of Intravenous BCG in Guinea-pigs and Pertinence to Cancer Immunotherapy in Man. *Lancet*, i, 1353.

PINSKY, C., HIRSHAUT, Y. & OETTGEN, H. (1972) Treatment of Malignant Melanoma by Intratumoral Injection of BCG. *Proc. Am. Assoc. Cancer Res.*, **13**, 21.

ROBINSON, E., BARTAL, A., COHEN, Y. & HAASZ, R. (1975) A Preliminary Report on the Effects of Methanol Extraction Residue of BCG (MER) on Cancer Patients. *Br. J. Cancer*, **32**, 1.

ROBINSON, E., BARTAL, A., COHEN, Y., HAASZ, R. & MEKORI, T. (1977) Treatment of Lung Cancer by Radiotherapy, Chemotherapy and Methanol Extraction Residue of BCG (MER): Clinical and Immunological Studies. *Cancer, N.Y.* (in press).

SPARKS, F. C., SILVERSTEIN, M. J., HUNT, J. S., HASKELL, C. M., PILCH, Y. H. & MORTON, D. L. (1973) Complications of BCG Immunotherapy in Patients with Cancer. *New Engl. J. Med.*, **289**, 827.